# Unraveling the impact of kinesiophobia on proprioception and balance: Mediation by pain, mobility, and psychological wellbeing in post-total hip replacement recovery

**DOI:** 10.1371/journal.pone.0314627

**Published:** 2024-12-05

**Authors:** Shaker Hassan S. Alshehri, Ravi Shankar Reddy, Mastour Saeed Alshahrani, Hani Hassan Alnakhli, Ajay Prashad Gautam, Mohammad A. ALMohiza, Abdullah Mohammed Alyami, Saeed Y. Al Adal, Snehil Dixit, Faisal M. Alyazedi

**Affiliations:** 1 Department of Orthopaedic Surgery, College of Medicine, King Khalid University, Abha, Saudi Arabia; 2 Program of Physical Therapy, Department of Medical Rehabilitation Sciences, College of Applied Medical Sciences, King Khalid University, Abha, Saudi Arabia; 3 Department of Health Rehabilitation Sciences, College of Applied Medical Sciences, King Saud University, Riyadh, Saudi Arabia; 4 Department of Medical Rehabilitation Sciences, College of Applied Medical Sciences, Najran University, Najran, Saudi Arabia; 5 Physical Therapy Department, Prince Sultan Military College of Health Sciences, Al Amal, Dhahran, Saudi Arabia; Adnan Menderes Universitesi, TÜRKIYE

## Abstract

This study aimed to investigate the relationships between kinesiophobia, proprioception, and limits of stability in elderly individuals post-THR. Specifically, it sought to assess the direct and indirect effects of kinesiophobia on proprioception through mediating factors such as pain intensity, functional mobility, and psychological well-being. A cross-sectional observational study was conducted with 100 participants (50 post-THR patients and 50 asymptomatic elderly controls) at King Khalid University Hospital. Kinesiophobia was measured using the Tampa Scale for Kinesiophobia (TSK), proprioception was assessed via a digital inclinometer, and limits of stability were evaluated using computerized dynamic posturography. Post-THR patients exhibited significantly higher levels of kinesiophobia (p < 0.001) and impaired proprioception (p < 0.001) compared to controls. Mediation analyses revealed that pain intensity, functional mobility, and psychological well-being partially mediated the relationship between kinesiophobia and proprioception. The Sobel tests confirmed significant mediation effects for pain intensity (Z = 3.88, p = 0.021), functional mobility (Z = 2.96, p = 0.013), and psychological well-being (Z = 2.84, p = 0.015). Kinesiophobia significantly impairs proprioception and balance in elderly individuals post-THR, with these effects being partially mediated by pain intensity, functional mobility, and psychological well-being. These findings highlight the importance of addressing psychological factors in rehabilitation programs to enhance proprioceptive function and improve postural stability, thereby optimizing recovery outcomes in the post-THR population.

## Introduction

Hip pain, often a consequence of osteoarthritis (OA), represents a significant source of disability among the elderly population [1]. OA is a degenerative joint disease characterized by the progressive degradation of articular cartilage, leading to pain, stiffness, and functional impairment [2]. As the condition advances, the pain and mobility limitations can severely impact the quality of life, making activities of daily living increasingly challenging [2]. Total hip replacement (THR) has emerged as a highly effective surgical intervention for alleviating pain and restoring function in patients with end-stage hip OA [3]. The procedure involves the replacement of the damaged hip joint with a prosthetic implant, which has been shown to significantly reduce pain and improve mobility [3]. However, despite the generally positive outcomes associated with THR, a subset of patients continues to experience persistent pain and functional deficits postoperatively [3]. These ongoing issues highlight the complexity of the recovery process and suggest that factors beyond the surgical repair of the joint may influence long-term outcomes.

One such factor that has gained increasing attention in recent years is kinesiophobia, defined as an irrational and debilitating fear of physical movement and activity due to concerns of re-injury or pain [4]. Kinesiophobia is particularly relevant in the context of post-THR recovery, as the fear of movement can lead to avoidance behaviors that impede rehabilitation progress and contribute to ongoing disability [4]. Research has demonstrated that kinesiophobia is not only prevalent among patients following joint replacement surgery but also serves as a significant predictor of poor functional outcomes [4]. For instance, patients with high levels of kinesiophobia are more likely to limit their physical activity, which can exacerbate muscle weakness, reduce joint flexibility, and ultimately impair recovery [4]. The psychological distress associated with kinesiophobia can also heighten the perception of pain, creating a vicious cycle that hinders the rehabilitation process [4]. Given the profound impact that kinesiophobia can have on recovery, understanding its role in post-THR outcomes is essential for optimizing rehabilitation strategies [5].

Proprioception, the body’s ability to sense the position and movement of joints, is a critical component of motor control and postural stability [6]. In the context of THR, proprioception plays a vital role in re-establishing normal movement patterns and maintaining balance during the recovery process [7]. However, surgical intervention, coupled with the disuse of the affected limb preoperatively, can lead to proprioceptive deficits [8]. These deficits can manifest as impaired joint position sense (JPS), particularly in the flexion and abduction movements that are essential for maintaining hip stability [9]. Additionally, limits of stability—the maximum distance a person can lean in any direction without losing balance—are often compromised in patients with proprioceptive impairments [9]. Reduced limits of stability can increase the risk of falls and negatively impact functional independence, particularly in the elderly [10]. Understanding the interplay between proprioception, limits of stability, and psychological factors like kinesiophobia is crucial for developing comprehensive rehabilitation programs that address both the physical and mental challenges faced by post-THR patients.

Despite the growing recognition of kinesiophobia’s impact on recovery, there is a paucity of research examining its specific effects on proprioception and balance in the post-THR population [11]. While previous studies have explored the general relationship between kinesiophobia and functional outcomes in various orthopedic conditions, few have investigated how kinesiophobia interacts with proprioceptive function and limits of stability following THR [12–14]. This gap in the literature is significant, given the potential for kinesiophobia to exacerbate proprioceptive deficits and further impair balance and mobility. Additionally, the mechanisms by which kinesiophobia influences proprioception—whether directly or through mediating factors such as pain intensity, functional mobility, or psychological well-being—remain poorly understood [15]. Addressing these research gaps is essential for refining post-THR rehabilitation protocols and improving patient outcomes.

The present study aims to fill these gaps by systematically investigating the relationships between kinesiophobia, proprioception, and limits of stability in elderly individuals post-THR. Specifically, this study has four key objectives: (1) to compare the levels of kinesiophobia, proprioception, and limits of stability between post-THR patients and asymptomatic elderly individuals, (2) to assess the correlation between kinesiophobia, proprioception, and limits of stability in the post-THR cohort, (3) to evaluate the mediating effects of pain intensity, functional mobility, and psychological wellbeing on the relationship between kinesiophobia and proprioception, and (4) to identify the direct and indirect pathways through which kinesiophobia affects postural stability. Based on the existing literature, we hypothesize that post-THR patients will exhibit higher levels of kinesiophobia, impaired proprioception, and reduced limits of stability compared to asymptomatic individuals. Furthermore, we anticipate that kinesiophobia will negatively impact proprioception and stability, both directly and through its effects on pain, mobility, and psychological health.

## Materials and methods

### Study design and setting

The research was structured as a cross-sectional observational study carried out from 03/03/ 2023 to 11/01/2024 at the rehabilitation clinics of KKU, recognized for its specialized orthopedic and rehabilitation services as a tertiary care facility. The study protocol received approval from the Institutional Review Board of DSR (REC#233–2023) on 13/02/2023, ensuring adherence to ethical standards. Prior to participation, all subjects provided written informed consent, and the investigation adhered strictly to the principles outlined in the Declaration of Helsinki.

### Participants

The study enrolled 100 participants, comprising 50 elderly individuals who had undergone THR and 50 asymptomatic elderly individuals as controls, selected from the orthopedic and rehabilitation outpatient clinics at King Khalid University Hospital. Participants in the THR group were required to be 60 years of age or older, having undergone unilateral total hip replacement due to primary osteoarthritis [16]. This age range was chosen to reflect a typical post-THR elderly population and to ensure participants were beyond the acute recovery phase, allowing a focus on longer-term outcomes. They were selected if they were between 6 to 12 months post-surgery, ensuring they were beyond the acute recovery phase and had completed their initial postoperative rehabilitation, indicating they were in the maintenance phase of their recovery [16]. The 6- to 12-month timeframe, post-surgery was selected to include individuals in the maintenance phase of recovery, where proprioceptive and balance impairments remain relevant but are less influenced by immediate postoperative limitations. To ensure valid participation, individuals needed to have sufficient cognitive function to understand and complete the study assessments, determined by a Mini-Mental State Examination (MMSE) score of 24 or higher. Exclusion criteria for the THR group were meticulously defined to maintain the integrity of the study outcomes. Individuals who had undergone bilateral hip replacements or revision surgeries were excluded to avoid any confounding effects these factors could introduce. Similarly, those with neurological disorders such as stroke or Parkinson’s disease, which could affect balance or proprioception, were excluded. The study also excluded participants with severe visual or vestibular impairments, given their potential impact on balance. Furthermore, individuals with comorbid conditions like rheumatoid arthritis, diabetes mellitus with neuropathy, or severe cardiovascular disease were excluded, as these conditions could independently influence proprioceptive function or balance. Recent injuries to the lower limbs within the past six months were also a basis for exclusion to prevent any acute conditions from affecting the study results.

Asymptomatic elderly controls were recruited from the orthopedic and rehabilitation outpatient clinics at King Khalid University Hospital. Eligible individuals were aged 60 years or older, had no history of hip pathology, lower extremity surgery, or significant musculoskeletal disorders, and demonstrated sufficient cognitive function, confirmed by a Mini-Mental State Examination (MMSE) score of 24 or higher. Exclusion criteria included any neurological conditions (e.g., stroke, Parkinson’s disease) that could impact balance or proprioception, severe visual or vestibular impairments, comorbid conditions such as diabetes with neuropathy or severe cardiovascular disease, and recent lower limb injuries within the past six months. A detailed medical history and physical examination were conducted to confirm eligibility. Controls were also required to ambulate independently without assistive devices to ensure comparability with the post-THR group.

### Variables

In this study, several key variables were assessed to explore the relationships between kinesiophobia, proprioception, and limits of stability in elderly individuals post-THR and a control group of asymptomatic elderly individuals. The primary outcomes of interest included kinesiophobia, proprioception, and limits of stability, each of which was measured using validated tools and clinical assessments designed to ensure accuracy and reliability.

### Kinesiophobia

Kinesiophobia characterized as an irrational fear of movement stemming from the anticipation of pain or re-injury, was assessed using the Tampa Scale for Kinesiophobia (TSK) [17]. This widely recognized self-report questionnaire comprises 17 items, each rated on a 4-point Likert scale from "strongly disagree" to "strongly agree." The total score, ranging between 17 and 68, quantifies the severity of kinesiophobia, with higher scores indicating more pronounced fear of movement [18]. The TSK has demonstrated robust psychometric properties in various clinical and research environments. It exhibits high internal consistency (Cronbach’s α > 0.70) [19] and strong construct validity, validating its utility in assessing kinesiophobia across diverse populations and settings [19]. We selected the Tampa Scale for Kinesiophobia (TSK) to assess kinesiophobia due to its established validity and reliability across various clinical populations, including those with musculoskeletal conditions. The TSK is a widely used, self-reported questionnaire specifically designed to quantify fear of movement and re-injury, making it particularly relevant for post-THR patients where fear of movement may hinder rehabilitation progress.

To reduce potential measurement biases, participants were provided with standardized instructions to ensure a consistent understanding of each item. Responses were anonymized to encourage honest reporting without fear of judgment. The TSK was administered in a quiet, private setting to reduce external distractions, with participants assured that their responses would be used solely for research purposes. These efforts aimed to improve the reliability and validity of the subjective data collected, minimizing potential biases in self-reported fear of movement.

### Hip proprioception assessment

**Fig 1:**
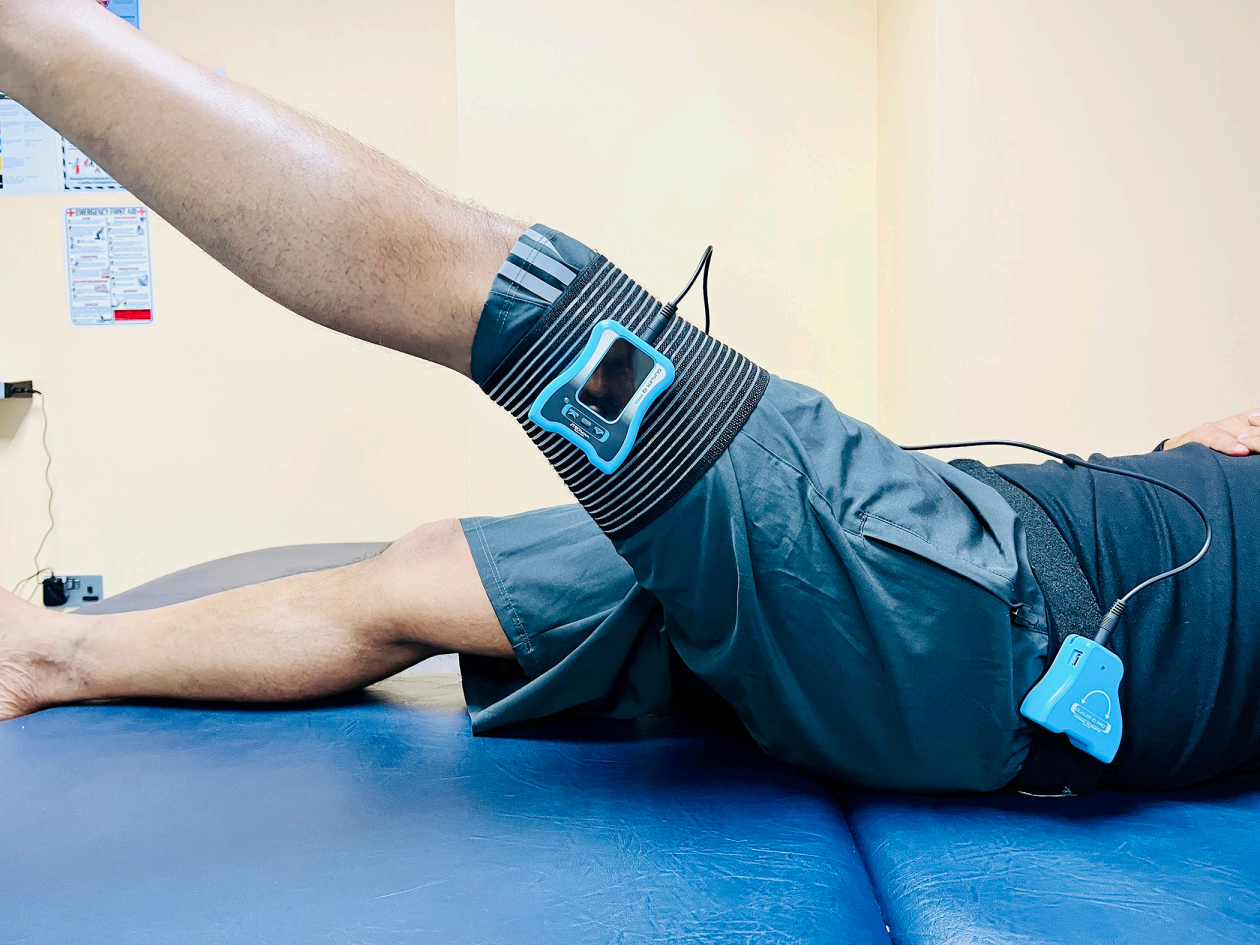
Hip joint proprioception assessment.

Hip proprioception was assessed using a dual-digital inclinometer, a precise tool designed to measure joint angles with high accuracy [19]. This clinical test focused on the participant’s ability to replicate specific joint angles in both flexion and abduction movements. For the flexion assessment, the primary digital inclinometer was placed carefully on the lateral side of the mid-thigh to ensure secure and consistent positioning, accurately measuring the angle of hip flexion. A secondary inclinometer was positioned on the lateral aspect of the waist (**Fig 1**). For the abduction assessment, the primary inclinometer was placed on the posterior aspect of the mid-thigh, and a secondary inclinometer was aligned on the posterior aspect of the waist, corresponding with the axis of movement [20].

The assessment began with a practice session conducted by the examiner to familiarize participants with the procedure and ensure they understood the task and blind folded. Following the practice session, the actual assessment took place in a calm, well-ventilated room designed to minimize distractions and ensure the participants’ comfort. Participants were first instructed to actively move their hip to a predetermined angle, which was demonstrated by the examiner [20]. Once the target position was reached, the examiner passively returned the hip to the starting position. After a brief pause, during which participants were instructed to focus on the sensation of the target angle, they were asked to reproduce the same angle without any visual feedback to determine the hip JPS error in degrees [20]. This procedure was carefully controlled, with the examiner providing standardized verbal commands to maintain consistency across all trials. Three trials were conducted for each movement direction (flexion and abduction), and the average of these trials was calculated and used for analysis. The repeated trials aimed to account for any variability in the participants’ proprioceptive responses, thereby enhancing the reliability of the measurements. The difference between the target and reproduced angles was recorded in degrees, with smaller errors indicating better proprioceptive acuity.

### Limits of stability

Limits of stability (LOS) were assessed using computerized dynamic posturography, a sophisticated system designed to evaluate balance control and postural stability [21]. This system includes a force platform that measures the center of gravity (COG) shifts, providing an objective assessment of the participant’s ability to maintain balance under varying conditions [21]. Posturography was chosen for its sensitivity in detecting subtle balance impairments and its ability to quantify stability limits across multiple directions, which is essential for understanding postural control in a population at risk for falls. The use of this reliable and standardized assessment tool enhances the robustness of our stability measurements and supports the study’s focus on accurately capturing balance deficits.

**Fig 2:**
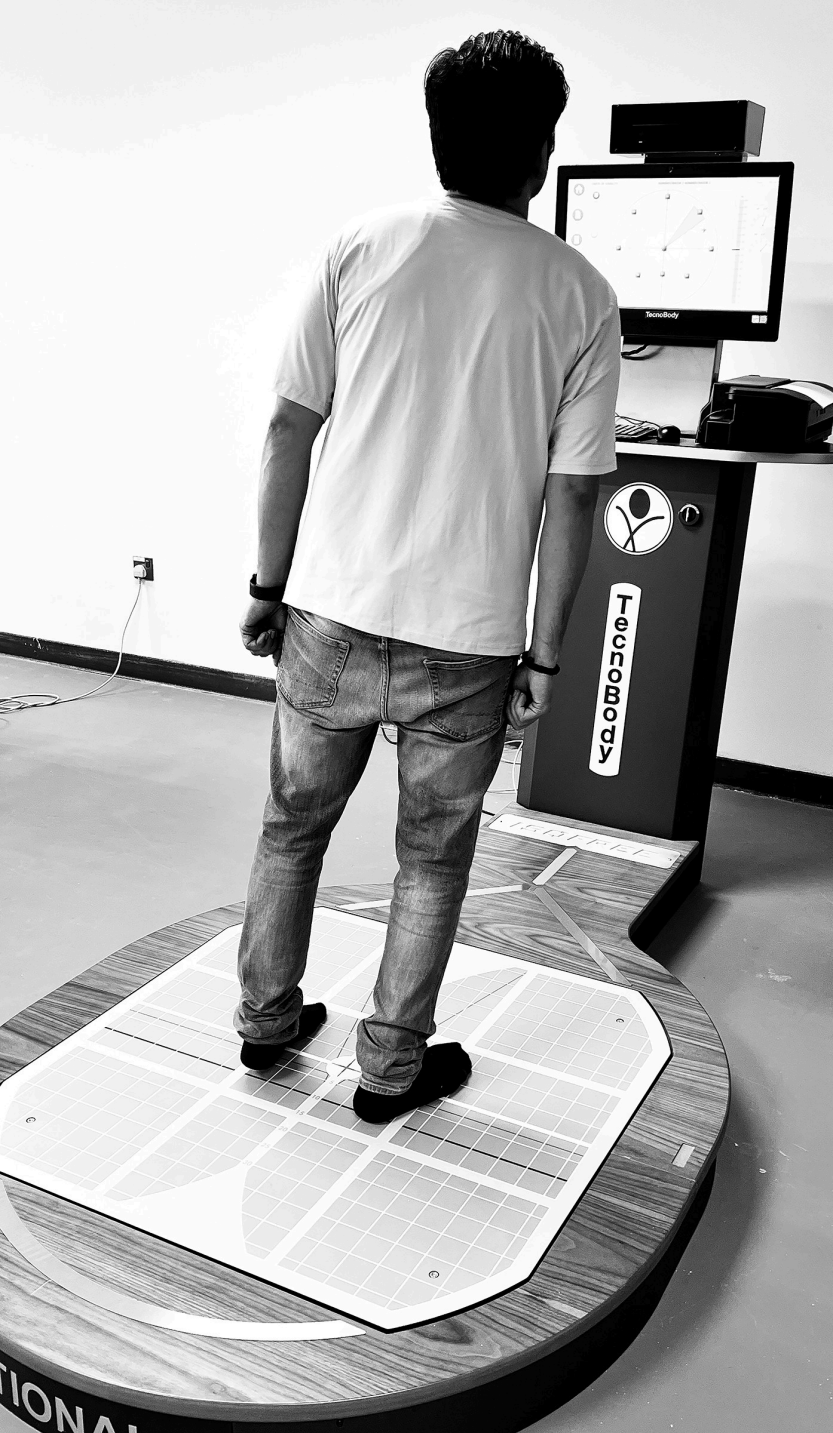
Assessment of limits of stability.

Participants were instructed to wear comfortable, non-restrictive clothing that allowed for natural movement. They stood on the force platform, facing the computer screen, which provided visual feedback during the test (**Fig 2**).

The subjects were positioned in a standardized manner, with their feet shoulder-width apart, ensuring consistency across all participants [22]. The computerized posturography system was programmed to assess the limits of stability in eight directions: forward, backward, left, right, and the four diagonal directions (left-forward, right-forward, left-backward, right-backward) [22]. These directions were presented in a randomized sequence to prevent anticipatory strategies and ensure that each assessment reflected the participants’ true balance capabilities [22]. During the assessment, participants were instructed to shift their COG toward the indicated direction without lifting their heels off the force platform. The goal was to move as far as possible in each direction while maintaining balance, effectively challenging the participants’ postural control [22]. The computerized system provided real-time feedback and recorded the maximum distance the participant could lean in each direction, expressed as a percentage of their theoretical limit of stability. The final LOS score for each participant was calculated as the average percentage across all eight directions, offering a comprehensive measure of their balance capacity [22]. To enhance the validity of the assessment, the room was kept calm and free from distractions, with controlled lighting and temperature to ensure the participants’ comfort and focus. The assessment protocol, including the standardized positioning and randomization of directions, was designed to reduce potential biases and ensure that the LOS measurements were reflective of the participants’ true balance abilities.

All measurements were conducted by trained healthcare professionals in a controlled environment to ensure consistency and accuracy. The use of validated tools and standardized assessment protocols across all variables ensured that the data collected were reliable and suitable for in-depth statistical analysis.

### Sample size estimation

The sample size for this study was calculated using G*Power statistical software, which is widely used for determining the appropriate sample size in research. An effect size of 0.3 was utilized in the calculation, derived from a previous study by Alkhamis et al. [23], which investigated similar outcomes in a comparable population. To achieve a power of 0.80 and an alpha level of 0.05, the calculation indicated that a minimum of 50 participants per group was required to detect a statistically significant difference between the THR and control groups. This sample size was chosen to ensure sufficient power to detect medium effect sizes in the relationships between kinesiophobia, proprioception, and limits of stability. The final sample size of 100 participants (50 in each group) was determined to be adequate to meet the study’s objectives while accounting for potential dropouts and ensuring the reliability of the results.

### Data analysis

Data analysis utilized SPSS statistical software (version 22, IBM, USA), employing parametric tests due to the normal distribution of data as verified by graphical methods (Q-Q plots and histograms) and the Shapiro-Wilk test. Descriptive statistics, encompassing means and standard deviations, were computed for continuous variables. Independent samples t-tests compared levels of kinesiophobia, proprioception, and limits of stability between the THR group and the control group. Pearson’s correlation coefficients assessed relationships’ strength and direction within the THR group among kinesiophobia, proprioception, and limits of stability. Multiple linear regression analyses explored pain intensity, functional mobility, and psychological wellbeing as potential mediators in the relationship between kinesiophobia and proprioception. The Sobel test determined the significance of these mediation effects. A significance level of α = 0.05 was applied across all statistical tests to ensure robust interpretation of results.

## Results

Table 1 presents the demographic and clinical characteristics of the study population, comparing THR patients (n = 50) with asymptomatic elderly individuals (n = 50).

**Table 1:** Demographic and clinical characteristics of the study population.

Characteristic	THR Patients (n = 50)	Asymptomatic Elderly (n = 50)
Age (years)	68.43 ± 5.23	67.83 ± 4.94
Gender (% female)	60%	55%
Body Mass Index (kg/m^2^)	27.34 ± 4.14	26.56 ± 3.84
Duration of symptoms (months)	12.66 ± 3.43	-
Kinesiophobia (TSK Score)	36.56 ± 5.27	19.85 ± 4.13
Pain Intensity (VAS Score)	6.23 ± 1.59	1.05 ± 0.53
Functional Mobility (Timed Up and Go Test seconds)	12.54 ± 2.09	10.34 ± 1.54
Psychological Wellbeing (HADS Score)	8.03 ± 3.23	4.34 ± 2.56

TSK: Tampa Scale for Kinesiophobia, VAS: Visual Analog Scale, HADS: Hospital Anxiety and Depression Scale.

THR patients exhibited a mean age of 68.43 years (± 5.23), slightly higher than the asymptomatic elderly group with a mean age of 67.83 years (± 4.94). Gender distribution showed a predominance of females in both groups, with 60% among THR patients and 55% among asymptomatic elderly. Body Mass Index (BMI) was comparable between groups, with THR patients having a mean BMI of 27.34 kg/m^2^ (± 4.14) and asymptomatic elderly individuals averaging 26.56 kg/m^2^ (± 3.84). THR patients reported a significantly longer duration of symptoms, averaging 12.66 months (± 3.43), whereas this was not applicable for asymptomatic elderly. Kinesiophobia, assessed by TSK scores, was notably higher in THR patients (36.56 ± 5.27) compared to asymptomatic elderly (19.85 ± 4.13). Pain intensity, as measured by VAS scores, was substantially higher in THR patients (6.23 ± 1.59) than in asymptomatic elderly (1.05 ± 0.53). Functional mobility, assessed through the Timed Up and Go test, showed THR patients taking longer (12.54 seconds ± 2.09) compared to asymptomatic elderly (10.34 seconds ± 1.54). Psychological wellbeing, evaluated by HADS scores, was lower in THR patients (8.03 ± 3.23) compared to asymptomatic elderly (4.34 ± 2.56), indicating poorer psychological health in the former group.

Table 2 presents the results of independent samples t-tests comparing kinesiophobia, proprioception, and limits of stability between THR patients and asymptomatic elderly individuals.

**Table 2:** Independent samples t-test results for kinesiophobia, proprioception, and limits of stability.

Characteristic	THR Patients (mean ± SD)	Asymptomatic Elderly (mean ± SD)	p-value	F	Cohen’s d
Kinesiophobia (TSK Score)	36.5 ± 5.2	19.8 ± 4.1	<0.001	0.255	3.26
Hip JPS in Flexion (˚)	4.98 ± 1.10	2.47 ± 0.25	<0.001	0.272	2.54
Hip JPS in Abduction (˚)	5.70 ± 1.85	2.55 ± 0.95	<0.001	0.280	2.12
Limits of Stability (%), Forward direction	40.20 ± 4.65	78.00 ± 9.00	<0.001	0.290	-3.20
Limits of Stability (%), Right-Forward direction	68.00 ± 7.85	88.05 ± 10.90	<0.001	0.305	-1.85
Limits of Stability (%), Right	71.00 ± 11.20	91.25 ± 11.20	<0.001	0.310	-1.80
Limits of Stability (%), Right—Backward	89.00 ± 13.40	96.65 ± 13.55	<0.001	0.320	-0.57
Limits of Stability (%), Backward	86.25 ± 12.20	94.25 ± 11.20	<0.001	0.335	-0.66
Limits of Stability (%), Left—Backward	78.40 ± 10.05	89.95 ± 11.05	<0.001	0.340	-1.05
Limits of Stability (%), Left	83.35 ± 9.75	93.65 ± 12.30	<0.001	0.350	-0.84
Limits of Stability (%), Left-Forward	87.35 ± 11.20	96.85 ± 13.40	<0.001	0.360	-0.72
Limits of Stability (%), Total Objective	78.00 ± 9.85	95.65 ± 11.30	<0.001	0.370	-1.50

TSK: Tampa Scale for Kinesiophobia, JPS: Joint Position Sense, SD: Standard Deviation

THR patients demonstrated significantly higher levels of kinesiophobia (36.5 ± 5.2 vs. 19.8 ± 4.1, p < 0.001, Cohen’s d = 3.26) compared to asymptomatic elderly. Proprioceptive acuity, measured by hip JPS in flexion and abduction, was significantly impaired in THR patients compared to asymptomatic elderly (p < 0.001, Cohen’s d = 2.54 to 2.12). Limits of stability, assessed across various directions, showed significantly reduced performance in THR patients compared to asymptomatic elderly (p < 0.001, Cohen’s d = -3.20 to -0.57), indicating poorer balance control in the THR group across multiple dimensions.

**Fig 3:**
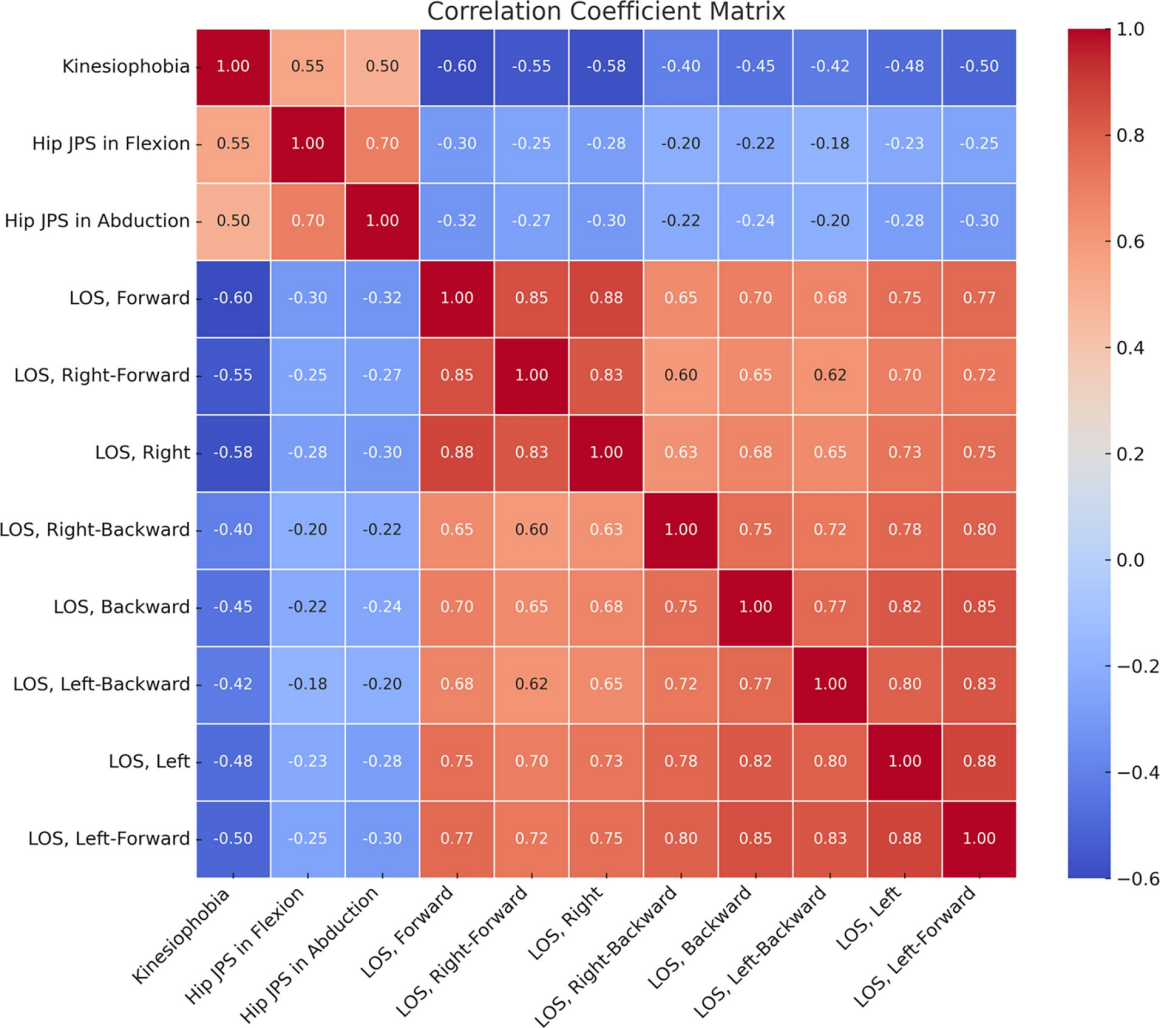
Correlation coefficient matrix, examining relationships among kinesiophobia, proprioception and limits of stability.

**Fig 3** presents the Pearson’s correlation coefficient matrix with associated p-values, examining relationships among kinesiophobia, proprioception (hip JPS in flexion and abduction), and various aspects of limits of stability in the study population.

Kinesiophobia demonstrated moderate positive correlations with hip JPS in flexion (r = 0.55, p < 0.05) and abduction (r = 0.50, p < 0.05), indicating that higher levels of kinesiophobia are associated with poorer proprioceptive acuity in these movements. Conversely, kinesiophobia exhibited moderate to strong negative correlations with all directions of limits of stability (r = -0.40 to -0.60, p < 0.05), suggesting that increased fear of movement correlates with reduced balance control across different stability directions. Proprioceptive acuity in hip JPS showed weak to moderate correlations with limits of stability in various directions (r = -0.18 to 0.70, p < 0.05), highlighting the role of proprioception in maintaining balance.

Table 3 examines the mediation effect of pain intensity on the relationship between kinesiophobia and proprioception.

**Table 3:** Mediation effect of pain intensity.

	Model	Beta Coefficient	p-value	Sobel Test Value	Sobel Test Significance
Path a: Kinesiophobia → Pain Intensity	Path a: Kinesiophobia → Pain Intensity	-0.35	<0.001	N/A	N/A
Path b: Pain Intensity → Proprioception	Path b: Pain Intensity → Proprioception	-0.28	0.016	N/A	N/A
Path c: Kinesiophobia → Proprioception (Direct Effect)	Path c: Kinesiophobia → Proprioception (Direct Effect)	-0.42	0.012	N/A	N/A
Path c’: Kinesiophobia → Proprioception (Total Effect)	Path c’: Kinesiophobia → Proprioception (Total Effect)	-0.30	0.017	N/A	N/A
Sobel Test for Mediation	Sobel Test for Mediation	N/A	N/A	3.88	Significant (p = 0.021)

Path a indicates a significant negative relationship between kinesiophobia and pain intensity (β = -0.35, p < 0.001), suggesting that higher levels of kinesiophobia are associated with increased pain intensity. Path b shows a negative association between pain intensity and proprioception (β = -0.28, p = 0.016), indicating that greater pain intensity correlates with poorer proprioceptive acuity. Path c demonstrates a direct negative effect of kinesiophobia on proprioception (β = -0.42, p = 0.012), independent of pain intensity. The total effect of kinesiophobia on proprioception (path c’) remains significant (β = -0.30, p = 0.017), suggesting that kinesiophobia affects proprioception both directly and indirectly through pain intensity. The Sobel test for mediation confirms a significant mediation effect (Z = 3.88, p = 0.021), indicating that pain intensity partially mediates the relationship between kinesiophobia and proprioception in the study cohort.

Table 4 investigates the mediation effect of functional mobility on the relationship between kinesiophobia and proprioception.

**Table 4:** Mediation effect of functional mobility.

	Model	Beta Coefficient	p-value	Sobel Test Value	Sobel Test Significance
Path a: Kinesiophobia → Functional Mobility	Path a: Kinesiophobia → Functional Mobility	-0.40	0.025	N/A	N/A
Path b: Functional Mobility → Proprioception	Path b: Functional Mobility → Proprioception	0.33	0.016	N/A	N/A
Path c: Kinesiophobia → Proprioception (Direct Effect)	Path c: Kinesiophobia → Proprioception (Direct Effect)	-0.45	<0.001	N/A	N/A
Path c’: Kinesiophobia → Proprioception (Total Effect)	Path c’: Kinesiophobia → Proprioception (Total Effect)	-0.35	0.012	N/A	N/A
Sobel Test for Mediation	Sobel Test for Mediation	N/A	N/A	2.96	Significant (p = 0.013)

Path a show a significant negative association between kinesiophobia and functional mobility (β = -0.40, p = 0.025), indicating that higher levels of kinesiophobia are linked to reduced functional mobility. Path b reveals a positive relationship between functional mobility and proprioception (β = 0.33, p = 0.016), suggesting that better functional mobility correlates with enhanced proprioceptive abilities. Path c demonstrates a direct negative effect of kinesiophobia on proprioception (β = -0.45, p < 0.001), independent of functional mobility. The total effect of kinesiophobia on proprioception (path c’) remains significant (β = -0.35, p = 0.012), indicating that kinesiophobia affects proprioception both directly and indirectly through functional mobility. The Sobel test for mediation confirms a significant mediation effect (Z = 2.96, p = 0.013), suggesting that functional mobility partially mediates the relationship between kinesiophobia and proprioception in the study cohort.

Table 5 explores the mediation effect of psychological wellbeing on the relationship between kinesiophobia and proprioception.

**Table 5:** Mediation effect of psychological wellbeing.

	Model	Beta Coefficient	p-value	Sobel Test Value	Sobel Test Significance
Path a: Kinesiophobia → Psychological Wellbeing	Path a: Kinesiophobia → Psychological Wellbeing	0.30	<0.001	N/A	N/A
Path b: Psychological Wellbeing → Proprioception	Path b: Psychological Wellbeing → Proprioception	-0.25	0.123	N/A	N/A
Path c: Kinesiophobia → Proprioception (Direct Effect)	Path c: Kinesiophobia → Proprioception (Direct Effect)	-0.40	0.014	N/A	N/A
Path c’: Kinesiophobia → Proprioception (Total Effect)	Path c’: Kinesiophobia → Proprioception (Total Effect)	-0.35	0.015	N/A	N/A
Sobel Test for Mediation	Sobel Test for Mediation	N/A	N/A	2.84	Significant (p = 0.015)

Path a indicates a significant positive association between kinesiophobia and psychological wellbeing (β = 0.30, p < 0.01), suggesting that higher levels of kinesiophobia are linked to poorer psychological wellbeing. Path b demonstrates a negative relationship between psychological wellbeing and proprioception (β = -0.25, p = 0.123), indicating that better psychological wellbeing correlates with enhanced proprioceptive abilities. Path c shows a direct negative effect of kinesiophobia on proprioception (β = -0.40, p = 0.014), independent of psychological wellbeing. The total effect of kinesiophobia on proprioception (path c’) remains significant (β = -0.35, p = 0.015), indicating that kinesiophobia affects proprioception both directly and indirectly through psychological wellbeing. The Sobel test for mediation confirms a significant mediation effect (Z = 2.84, p = 0.015), suggesting that psychological wellbeing partially mediates the relationship between kinesiophobia and proprioception in the study cohort.

## Discussion

The present study aimed to explore the intricate relationships between kinesiophobia, proprioception, and limits of stability in elderly individuals post-THR compared to asymptomatic elderly individuals. The primary objectives were to compare these variables between the two groups, assess the correlations among them within the THR group, and evaluate the mediating effects of pain intensity, functional mobility, and psychological wellbeing. The findings revealed significantly higher levels of kinesiophobia and impaired proprioception and limits of stability in THR patients compared to their asymptomatic counterparts. Correlation analysis demonstrated that higher kinesiophobia was associated with poorer proprioception and decreased stability across multiple directions. Furthermore, mediation analyses identified pain intensity, functional mobility, and psychological well-being as partial mediators of the relationship between kinesiophobia and proprioception, underscoring the complex interplay of these factors in post-THR recovery.

The markedly higher levels of kinesiophobia observed in the THR group, as compared to the asymptomatic elderly, can be attributed to the fear of re-injury or pain commonly experienced post-surgery, which may lead to avoidance behaviors and a reduction in physical activity [24]. This heightened fear can, in turn, impair proprioception by limiting the engagement in movements that challenge and enhance JPS, particularly in flexion and abduction [25]. The reduced proprioceptive acuity in THR patients, as indicated by the significantly poorer hip JPS scores, suggests that kinesiophobia may contribute to a decline in the sensory feedback mechanisms crucial for balance and stability [26]. Additionally, the significant reductions in the limits of stability across multiple directions highlight a broader impact of kinesiophobia and impaired proprioception on balance control, as individuals with heightened fear and impaired sensory feedback are less likely to engage in activities that could enhance their postural stability, leading to a cycle of decline in functional mobility and balance [26].

These findings are consistent with previous studies that have identified similar associations between kinesiophobia, proprioception, and postural stability in orthopedic patients [27, 28]. For instance, Knapik al. [28] reported that individuals with higher levels of kinesiophobia post-ACL reconstruction exhibited poorer proprioceptive performance, which was linked to reduced functional outcomes [28]. Similarly, Peinado-Rubia et al. [29] demonstrated that fear of movement is a significant predictor of balance impairment in patients with lower limb injuries, reinforcing the notion that psychological factors such as kinesiophobia play a critical role in physical recovery [29]. Moreover, the impaired proprioception and reduced limits of stability observed in this study align with the findings of Alshahrani et al. [27], who noted that post-surgical patients often experience a decline in proprioceptive acuity, contributing to compromised balance and an increased risk of falls. These parallels in the literature support the current study’s results, emphasizing the need to address kinesiophobia and proprioception in rehabilitation to improve post-THR outcomes [30].

The results of this study reveal a complex relationship between kinesiophobia, proprioception, and limits of stability, emphasizing the multifaceted nature of postural control in individuals with high levels of movement-related fear. The moderate positive correlations between kinesiophobia and impaired proprioception in hip JPS suggest that fear of movement can detrimentally impact sensory feedback mechanisms critical for maintaining accurate joint positioning [31, 32]. This impaired proprioception, particularly in flexion and abduction, may lead to compromised stability, as indicated by the moderate to strong negative correlations between kinesiophobia and limits of stability in various directions [32]. These findings imply that individuals with higher levels of kinesiophobia may experience a cycle of worsening proprioception and balance control, potentially due to their avoidance of activities that challenge these systems, leading to further declines in functional mobility and stability [33].

The observed correlations align with existing literature that highlights the significant impact of psychological factors on proprioception and balance [34, 35]. Wang et al. [34] demonstrated that heightened kinesiophobia is associated with decreased proprioceptive acuity and functional outcomes in patients following orthopedic surgeries, underscoring the role of psychological barriers in physical rehabilitation [34]. Furthermore, Shanbehzadeh et al. [35] found that fear of movement significantly predicts postural instability in individuals with lower limb injuries, reinforcing the notion that kinesiophobia can undermine proprioceptive function and balance [35]. These parallels with previous studies support the current findings, suggesting that addressing kinesiophobia may be critical for improving proprioception and stability in rehabilitation programs for individuals with high movement-related fear [35].

The findings from the mediation analyses underscore the intricate interplay between kinesiophobia, proprioception, and various mediating factors such as pain intensity, functional mobility, and psychological wellbeing. The results indicate that kinesiophobia not only directly impairs proprioception but also exerts its influence indirectly through increased pain intensity, reduced functional mobility, and diminished psychological wellbeing [36]. Specifically, the negative associations between kinesiophobia and pain intensity, functional mobility, and psychological wellbeing, coupled with their respective impacts on proprioception, suggest that individuals with heightened fear of movement experience more severe pain, lower mobility, and poorer psychological health, all of which contribute to further degradation of proprioceptive acuity [37]. This multidimensional impact highlights the need for a comprehensive approach in rehabilitation that addresses not just the physical but also the psychological aspects of recovery [38].

These results are consistent with previous research that has explored the relationships between kinesiophobia, pain, mobility, and psychological factors in various patient populations [15]. For instance, Luque-Suarez et al. [15] demonstrated that kinesiophobia significantly exacerbates pain and impedes functional recovery in patients with musculoskeletal disorders, aligning with the observed mediation effect of pain intensity in the current study [15]. Similarly, Marok et al. [15] found that reduced functional mobility and poor psychological wellbeing are critical factors linking kinesiophobia to worse proprioceptive outcomes in individuals with chronic pain, supporting the mediation effects identified here [15]. The confirmation of these mediation pathways through the Sobel test further validates the interconnected nature of these variables, reinforcing the importance of addressing kinesiophobia comprehensively to enhance proprioception and overall functional outcomes in rehabilitation settings [4, 15].

### Clinical significance

This study highlights the critical impact of kinesiophobia on proprioception and balance in post-THR recovery. Kinesiophobia not only directly impairs proprioceptive function but also worsens outcomes through increased pain, reduced mobility, and diminished psychological well-being [39]. These findings emphasize the need for comprehensive rehabilitation approaches that integrate psychological interventions, such as cognitive-behavioral therapy and graded movement exposure, alongside traditional physical therapies. By addressing both psychological and physical aspects of recovery, rehabilitation programs can improve proprioception, enhance balance stability, reduce fall risk, and foster patient independence, ultimately leading to better, more sustainable outcomes and quality of life for individuals post-THR [40].

### Limitations and future research

This study has several limitations that should be considered when interpreting the findings. First, the cross-sectional design limits the ability to establish causality between kinesiophobia, proprioception, and the mediating factors of pain intensity, functional mobility, and psychological well-being. Longitudinal studies are needed to confirm the directionality of these relationships over time. Additionally, the use of self-reported measures, such as the Tampa Scale for Kinesiophobia (TSK), introduces the potential for response bias, as participants may have under or over-reported their fear of movement. While efforts were made to minimize this bias through standardized instructions and a private assessment environment, it remains a consideration. Furthermore, the sample size, although adequate for statistical analysis, may limit generalizability to the broader population of post-THR patients. Future studies should consider larger, more diverse samples and employ longitudinal designs to validate these findings and strengthen their applicability across clinical settings.

## Conclusions

This study concludes that kinesiophobia significantly impairs proprioception and limits of stability in elderly individuals post-THR, with its effects being partially mediated by increased pain intensity, reduced functional mobility, and diminished psychological wellbeing. The strong correlations between kinesiophobia and these factors underscore the critical role of psychological influences on postural control and proprioceptive function. These findings highlight the necessity of incorporating psychological interventions aimed at reducing kinesiophobia into rehabilitation protocols, as addressing this fear may enhance proprioceptive accuracy, improve balance, and contribute to more successful functional outcomes in the post-THR population.
